# Observational study of intraocular lens tilt in sutureless intrascleral fixation versus standard transscleral suture fixation determined by ultrasound biomicroscopy

**DOI:** 10.1186/s40942-019-0182-y

**Published:** 2019-07-29

**Authors:** Bruna Ferraço Marianelli, Thaís Sousa Mendes, Roberta Pereira de Almeida Manzano, Patrícia Novita Garcia, Ivan Corso Teixeira

**Affiliations:** 1 Department of Ophthalmology, Santa Casa de São Paulo, São Paulo, Brazil; 2 Department of Ophthalmology, Federal University of São Paulo, São Paulo, Brazil

**Keywords:** Intraocular lens scleral fixation, Intraocular lens implantation, Ultrasound biomicroscopy, Lens subluxation, Cataract

## Abstract

**Background:**

The position of the intraocular lens (IOL) is a major factor that affects the final visual acuity after cataract surgery. However, no prospective study has compared the IOL positions associated with the sutureless intrascleral technique and the standard transscleral suturing technique. The current study compared the IOL positions in the two techniques using ultrasound biomicroscopy (UBM) in vivo.

**Methods:**

Twenty-one eyes of 21 patients were included in this observational study conducted between February and May 2015. Eleven patients underwent the sutureless intrascleral technique, and 10 patients underwent transscleral fixation with suturing. Ophthalmologic examination and UBM were performed in all patients. Optic tilt was measured in relation to the iris plane. The haptic location was defined. Mann–Whitney test and multiple linear regression were used to analyze the vertical and horizontal gradients. Significant differences were considered when p ≤ 0.05.

**Results:**

The most common indication for scleral fixation was a complication during phacoemulsification (81.81% in the sutureless group and 60% in the suture group). The mean vertical and horizontal tilts were, respectively, 0.24 ± 0.21 and 0.25 ± 0.19 mm in the sutureless group and 0.14 ± 0.17 and 0.23 ± 0.16 mm in the suture group. No significant differences were seen in the vertical tilt and horizontal tilt (p = 0.888 and p = 0.148, respectively) between the groups. Gender (p = 0.835), age (p = 0.888), follow-up time (p = 0.915), and surgical duration (p = 0.094) were not associated with optic tilt. Of the 22 haptics in the sutureless group, 21 (95.45%) were in the intrascleral tunnel; of the 20 haptics in the suture group, 13 (65%) were posterior to the ciliary body, four (20%) anterior to the ciliary body, and three (15%) in the ciliary sulcus.

**Conclusion:**

This study showed that there are no significant differences in the IOL positions between the two techniques.

## Background

A fundamental step in cataract surgery to optimize visual rehabilitation is implantation of an intraocular lens (IOL) after successful extraction of the crystalline lens [1]. The IOL is implanted in the capsular bag in most uncomplicated cases. However, when the capsular support is insufficient, the IOL can be implanted in the anterior chamber, fixated in the iris, or fixated in the ciliary sulcus by a transscleral suture [1–3]. These alternatives can result in complications; Anterior chamber IOLs can accelerate endothelial cell loss, corneal decompensation can occur, and recurrent uveitis may develop after implantation of iris-fixated IOLs [2, 4].

In 1997, Maggi and Maggi [5] described a sutureless technique for IOL scleral fixation, which Gabor and collaborators modified later [1]. The basic principle of this technique, which is common to most variations described in the literature, is incarceration of the IOL haptics in a scleral tunnel parallel to the limbus [1, 5].

The absence of a suture seems to reduce the surgical time and complications [2]. Long-term monitoring of patients who underwent conventional scleral fixation has identified suture-related complications, such as scleral and conjunctival erosion, suture-induced inflammation, suture degradation, and late luxation or subluxation of the IOL after suture rupture [6–8]. Those potential suture-related complications could be prevented by a sutureless technique of scleral fixation of IOLs.

The IOL position is a major factor that affects the final visual acuity (VA) after cataract surgery [9]. IOL tilt and decentration can cause defocusing, astigmatism, and wavefront aberrations postoperatively [10, 11]. In addition, adequate position prevents complication, such as the uveitis-glaucoma-hemorrhage syndrome, caused by prolonged contact between the haptics and uveal tissue [2, 3]. In the sutureless technique of scleral fixation of IOLs, a major part of the haptic remains incarcerated inside the scleral tunnel, which seems to stabilize the IOL and reduce the IOL tilt [1, 12].

The sutureless intrascleral technique can be performed with a standard foldable IOL, which eliminates the need to stock specific types of IOLs [1, 2]. Another advantage is that the technique is simple, without the need for complicated suture procedures [1, 3].

Ultrasound biomicroscopy (UBM) is a contact method that generates high-resolution images of anterior-segment structures and is considered the standard method for accessing the IOL position [13]. Previous studies have demonstrated the use of UBM for evaluating the postoperative IOL position fixated to the sclera [14].

The aim of this study was to compare the IOL tilt in both the sutureless intrascleral technique and the standard transscleral suturing technique using UBM to access the IOL position in vivo.

## Methods

The current study was a prospective and observational study conducted between February and May 2015. We selected patients from the Santa Casa de Misericórdia de São Paulo (SCMSP), Department of Ophthalmology, and the Suel Abujamra Institute (ISA) who underwent IOL scleral fixation surgery. Twenty-one eyes of 21 patients, 11 from SCMSP and 10 from ISA, were divided into two groups according to the surgical technique, i.e., the sutureless intrascleral group and the transscleral suture fixation group. One of two ophthalmologic surgeons from the Santa Casa de Misericórdia de São Paulo with the same level of surgical expertise performed all surgeries. Both surgeons performed a similar number of surgeries of the two techniques.

The exclusion criteria were a postoperative period shorter than 1 month, uncooperative patients, aniridia, ocular trauma with relevant disruption of the anterior-segment anatomy, a previous glaucoma or corneal surgery (except for refractive surgery), patients who did not agree to participate in the study and did not provide informed consent. The Research Ethic Committee of SCMSP approved the study, which adhered to the principles of the Declaration of Helsinki.

In the sutureless intrascleral technique, two straight sclerotomies ab externo were prepared with a 24-gauge cannula 1.50 to 2.00 mm from the limbus and 180 degrees apart. A tunnel parallel to the limbus then was created at about 50% scleral thickness, starting from the sclerotomies and ending with the externalization of the cannula after to 3.00 mm. A three-piece IOL was implanted using an injector. The leading haptic was grasped at the tip with an end-gripping 25-gauge forceps (Schariot IOL Scleral Fixation forceps 25 gauge, Dorc), pulled through the sclerotomy, and left externalized. The forceps was introduced into the distal end of the tunnel, the externalized tip grasped, and the IOL haptic pulled into the tunnel. The same process was repeated with the second haptic. The final steps were adjustment of the IOL position and centration.

In the sutured transscleral fixation technique, two triangular 3-mm scleral flaps were created 180 degrees apart with the base on the limbus. A 10-0 double-armed polypropylene suture with a straight needle then was introduced under the center of one of the scleral flaps 1.5 to 2 mm from the limbus, and the needle exited the eye at the center of the other flap, with the help of a 24-gauge cannula. The polypropylene suture was pulled outside the eye through the scleral sulcus, cut in two parts, and each part was tied to a haptic. After the IOL was implanted by scleral sulcus and centered, the transscleral sutures were placed under each scleral flap.

All patients underwent a complete ocular examination and UBM scan. One experienced physician performed the UBM scans using the VuMax II (Sonomed Escalon, New Hyde Park, NY, USA) with a 50-MHz transducer. The examination was performed under standard conditions with the room lights on, no pharmacologic mydriasis, and the patient in a supine position. Topical anesthesia was induced by proxymetacaine hydrochloride 5 mg/ml and an eyecup containing normal saline solution.

**Fig. 1:**
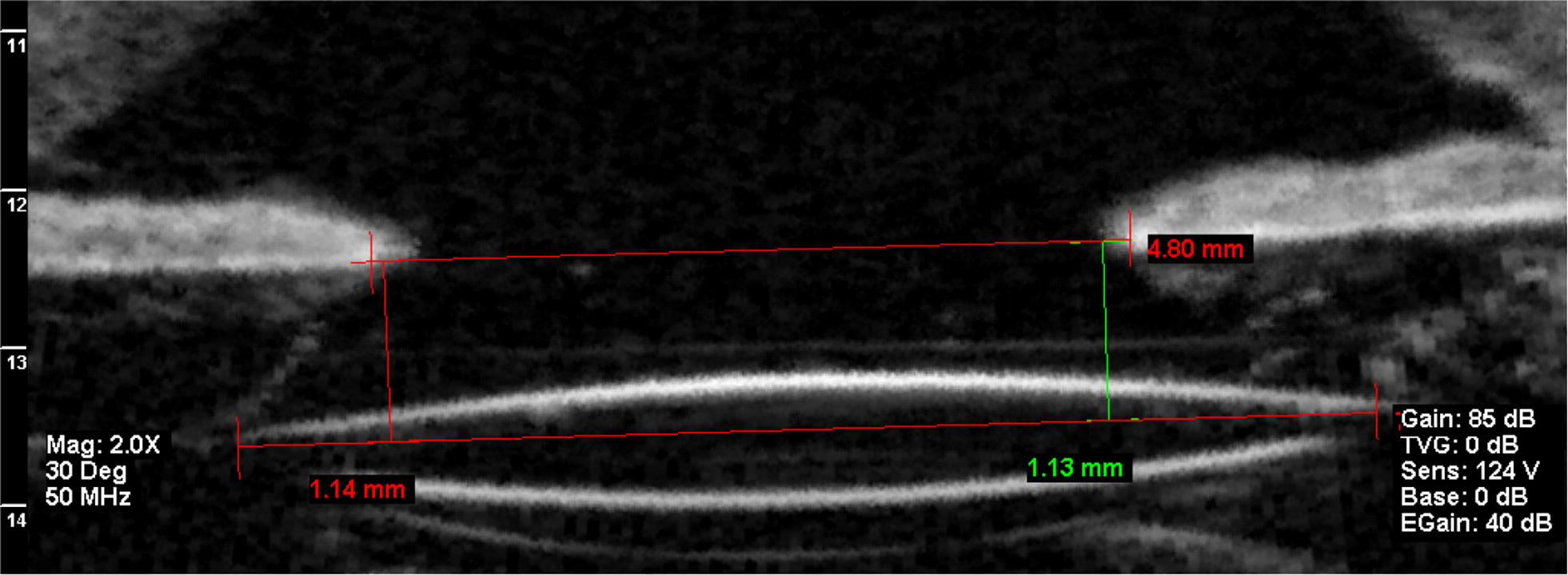
An ultrasound biomicroscopy image shows an axial vertical image of an eye that underwent the sutureless intrascleral fixation technique and the method used to measure the vertical tilt

**Fig. 2:**
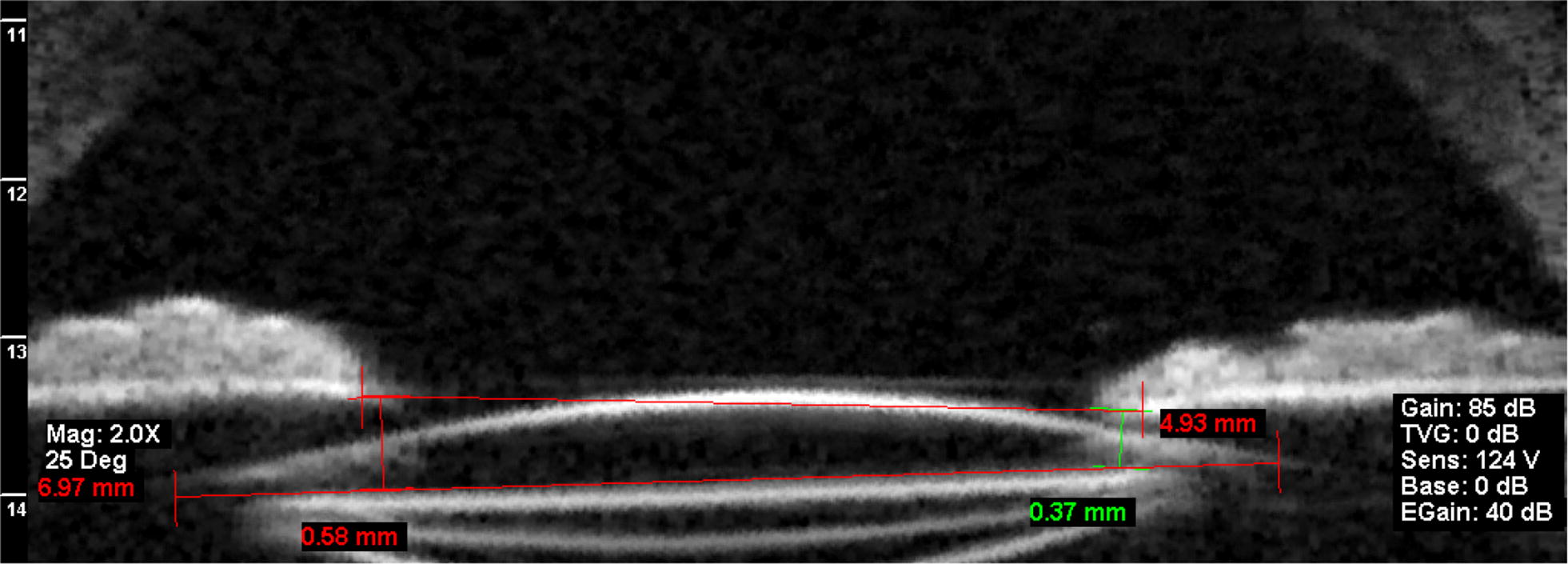
An ultrasound biomicroscopy image shows an axial horizontal image of an eye that underwent the sutured transscleral fixation technique showing the method used to measure the horizontal tilt

The focus of this study was determination of the IOL position in the posterior chamber. The IOL optic tilt was measured using the method of Loya et al. [15] as reported by Kumar et al. [9]. The first step involved drawing a line along the hyperreflective iris pigment epithelium and uniting the edges of the pupil, which was the plane of reference. A second line along the anterior face of the IOL optic then was drawn. Finally, the smallest distance between these two lines was measured using the caliper tool in the UBM system in specific positions: superior (12 clock hours), inferior (6 clock hours), medial (3 clock hours in the right eye, 9 clock hours in the left eye), and lateral (9 clock hours in the right eye, 3 clock hours in the left eye) (Figs. 1, 2). Since iris integrity was determinant to establish the IOL position measures, eyes with aniridia or relevant disruption of the anterior-segment (including iris) anatomy was excluded from this study.

The difference between the superior distance and inferior distance was defined as the vertical tilt; the difference between the medial distance and lateral distance was defined as the horizontal tilt. One physician performed all measurements according to the protocol described and revised by a second physician. If there was any disagreement between these two examiners, a third physician was consulted.

The haptic location was determined by observing the high reflection obtained from the haptic. In the sutureless intrascleral fixation group, we expected to find the haptic inside the scleral tunnel. In the sutured transscleral fixation group, we found the haptics in three positions, i.e., anterior to the ciliary body, posterior to the ciliary body, and in the ciliary sulcus.

The IOL types used in this study were the Sensar AR40e (n = 6) (Johnson & Johnson Vision, Santa Ana, CA, USA), Oft Vision CP72BU (n = 3) (Oft Vision, São Paulo, SP, Brazil) and Sofport (Bausch & Lomb, Rochester, NY, USA) (n = 2) in the sutureless group; in the suture group, all IOLs were Oft Vision PC700FE, except one, which was a Lite Fit E (Visiontech, Nova Lima, MG, Brazil). Oft Vision PC700FE is a PMMA single piece IOL, with 12.5 mm lenght and 6.5 mm optic-size, specific designed for scleral fixation.

Data were collected and stored in Excel tables (Microsoft Corp., Redmond, WA, USA). Continuous variables were expressed as the means and standard deviations, and categorical variables were expressed as absolute values and frequencies. Statistical analyses were performed using Stata/SE Statistical Software, Release 14.0, 2015 (Stata Corp, College Station, Texas, USA). The data normal distribution was evaluated using Shapiro–Wilk test. Numerical variables were compared between the two groups under study by the Mann–Whitney test. The associations between categorical variables were assessed through the Chi-Square test. Multiple linear regression was used to evaluate the association between continuous and categorical variables adjusting for covariates of interest. p values ≤ .05 were considered statistically significant.

## Results

Twenty-one eyes of 21 patients were analyzed. Eleven patients (6 women, 5 men; mean age, 65.18 ± 12.65 years) comprised the sutureless scleral fixation group. Ten patients (5 women, 5 men; mean age, 62.6 ± 21.33 years) comprised the standard transscleral suturing technique. The mean time from the surgical date to the UBM scan was 4.36 ± 3.23 months for the sutureless scleral fixation group and 4.3 ± 3.56 months for the transscleral suture group.

In both groups, the major indication for scleral fixation was the development of a complication during phacoemulsification, which occurred in 81.81% of the sutureless group and 60% of the suture group. Other indications included lens subluxation and ocular trauma (Table 1).

**Table 1:** Indications for scleral fixation surgery

Surgery indication	Sutureless intrascleral fixation (n = 11)	Transscleral suture fixation (n = 10)
Phacoemulsification complications	9 (81.81%)	6 (60%)
Lens subluxation	2 (18.18%)	1 (10%)
Ocular trauma	–	3 (30%)

In the sutureless and the suture groups, the mean vertical tilt values were 0.24 ± 0.21 mm and 0.14 ± 0.17 mm, respectively, and the respective mean horizontal tilt values were 0.25 ± 0.19 mm and 0.23 ± 0.16 mm. The differences in the IOL inclination were not significant for vertical tilt (p = 0.148) or horizontal tilt (p = 0.888) (Tables 2, 3) between the groups. Gender (p = 0.835), age (p = 0.888), follow-up time (p = 0.915), and surgical duration of (p = 0.094) were not associated with optic tilt.

**Table 2:** Vertical inclination

Technique	Sutured	Sutureless
Number (total)	10	11
Mean ± standard deviation	0.14 ± 0.17	0.24 ± 0.21
p value	0.148	

Vertical inclination mean ± standard deviation, standard error, 95% confidence intervals and *p* value for each technique

**Table 3:** Horizontal inclination

Technique	Sutured	Sutureless
Number (total)	10	11
Mean ± standard deviation	0.23 ± 0.16	0.25 ± 0.19
p value	0.888	

Horizontal inclination mean ± standard deviation, standard error, 95% confidence interval and *p* value for each technique

In the sutureless group, the mean distances between the iris pigment epithelium and the IOL optic were 0.92 ± 0.43 mm at 12 clock hours, 0.84 ± 0.41 mm at 6 clock hours, 0.87 ± 0.57 mm at 9 clock hours, and 0.90 ± 0.40 mm at 3 clock hours, and in the suture group 0.95 ± 0.66 mm at 12 clock hours, 1.06 ± 0.61 mm at 6 clock hours, 0.94 ± 0.70 mm at 9 clock hours, and 1.01 ± 0.47 mm at 3 clock hours. No significant differences were seen between the two groups for each of these four positions (p = 0.778 at 12 clock hours, p = 0.597 at 6 clock hours, p = 0.860 at 9 clock hours and p = 0.647 at 3 clock hours).

The variables of main interest “vertical tilt” and “horizontal tilt” did not show correlation with surgical technique (p > 0.05). The association analyzed by Mann–Whitney test, however, do not consider possible co-variables that may influence the relationship between tilt and group. Thus, a multiple linear regression adjusted for gender, age, surgical duration and follow-up time was made, and the results are showed in Table 4.

**Table 4:** Multiple linear regression

	Vertical tilt coefficient (95% confidence interval)	p	Horizontal tilt coefficient (95% confidence interval)	p
Technique				
Sutured	Reference	–	Reference	–
Sutureless	0.082 (− 0.112 to 0.277)	0.379	0.055 (− 0.126 to 0.236)	0.527
Gender				
Female	Reference	–	Reference	–
Male	− 0.105 (− 0.291 to 0.082)	0.248	− 0.157 (− 0.331 to 0.016)	0.072
Age	− 0.005 (− 0.011 to 0.001)	0.082	− 0.001 (− 0.006 to 0.005)	0.822
Surgical duration	− 0.001 (− 0.003 to 0.002)	0.580	0.001 (− 0.001 to 0.003)	0.163
Follow-up time	− 0.008 (− 0.038 to 0.021)	0.546	0.005 (− 0.022 to 0.032)	0.704

Twenty-one of 22 (95.45%) haptics in the sutureless group were positioned correctly in the intrascleral tunnel; one (4.54%) migrated to the ciliary sulcus. Of the 20 haptics in the suture group, 13 (65%) were posterior to the ciliary body, four (20%) were anterior to the ciliary body and three (15%) were in the ciliary sulcus.

The mean postoperative best corrected visual acuity (BCVA) was 0.45 ± 0.39 (logMAR) in the sutureless group and 0.55 ± 0.32 (logMAR) in the suture group (p = 0.439). There was no statistic significant differences for postoperative spheric error (p = 0.525), cylindrical error (p = 0.179) and spheric equivalent (p = 0.160) between the groups. Visual acuity results and postoperative refraction data are shown in Table 5.

**Table 5:** Visual acuity results and postoperative refraction

Technique	Sutured	Sutureless	p
	Mean ± standard deviation (median)	Mean ± standard deviation (median)	
Postoperative spheric error	− 0.21 ± 1.79 (− 0.50)	0.32 ± 2.15 (0.00)	0.525
Postoperative cylindrical error	− 3.36 ± 1.86 (− 2.50)	− 2.14 ± 1.03 (− 2.00)	0.179
Postoperative spheric equivalent	− 1.89 ± 1.64 (− 2.00)	− 0.75 ± 1.97 (− 0.25)	0.160
Postoperative best corrected visual acuity (logMAR)	0.55 ± 0.32 (0.48)	0.45 ± 0.39 (0.30)	0.439

Postoperative spheric and cylindrical error (diopters), spheric equivalent (diopters) and best corrected visual acuity (logMAR): mean ± standard deviation, median, and p value.

## Discussion

Cataract surgery has advanced markedly in the previous decades with the development of phacoemulsification and small-incision surgery with IOL implantation, which improved the postoperative results and reduced the complications. Following this, the primary concern has become the visual quality after cataract surgery. The IOL position has an important effect on the final VA results [9]. IOL tilt and decentration may result in defocusing, astigmatism, and wavefront aberrations postoperatively [10, 11]. Since cases of insufficient capsular support are not rare in daily practice and there is no consensus regarding which technique achieves superior IOL fixation, efforts are being directed toward this field of research.

In the current study, the major indications for sutureless intrascleral fixation of IOLs were complications that developed during phacoemulsification, which also were reported by Kumar et al. [2], and Scharioth et al. [3], that found that capsular defects during phacoemulsification were the most prevalent indication (56% and 25.40%, respectively). The disparity between the frequencies can be explained by the larger sample sizes of these studies, which included more rare conditions, e.g., Marfan syndrome and pseudoexfoliation syndrome. In addition, more surgical complications were expected to occur at SCMSP and ISA, because they are training centers for medical residents in Ophthalmology. Another important factor regarding the indication was that the sutureless and suture groups were heterogeneous, since patients who had undergone complicated phacoemulsification represented 81.81% of the sutureless group and 60% of the suture group, what may affect the IOL position. We attempted to minimize the possible effects of these differences between the groups by excluding eyes with substantial loss of anterior-segment anatomy and removing the capsular remnants in all cases.

We used three different IOLs for the sutureless technique: the Sensar AR40e, Oft Vision CP72BU, and Sofport. Schariot et al. [3] included these IOLs in a series of 63 cases using six types of lenses. The most frequently used IOL was the Sensar AR40e (60.32%). Kumar et al. [9] also reported using two different IOLs in a study of 46 eyes to determine the IOL position by UBM; one was a rigid IOL and the other a three-piece foldable IOL. Kumar et al. [9] concluded that there was no significant difference in the optic position between the two types of IOL.

In the current study, no significant differences were seen between the two groups when we compared the vertical and horizontal IOL tilt. Additionally, postoperative best corrected visual acuity and postoperative refraction results (spherical error, cylindrical error and spheric equivalent) showed no statistic significant differences between the surgical techniques. Because of the incarceration of a large portion of the IOL haptics inside the scleral tunnel parallel to the limbus, the sutureless technique is considered to offer greater IOL stability with reduced tilt compared to the conventional technique involving sutures [1, 3]. Despite the fact that some authors have reported this theoretical advantage [1–3, 9], to our knowledge, no study has compared the two techniques (sutureless intrascleral fixation versus standard transscleral suturing technique).

Similar studies in the literature have compared other techniques of IOL fixation. In 2016, Horiguchi et al. [16] compared the ab interno and ab externo IOL scleral fixation techniques, which are both variants of the sutured transscleral fixation, and concluded that both techniques achieved similar results in haptic placement, but the ab externo technique was associated with greater vertical tilt. In 2018, Chantarasorn et al. [17] compared reinforced scleral fixation using double sutures and intrascleral IOL fixation. That study was similar to the current study; the only subtle difference was in the transscleral suture technique, in which two sutures was used to fixate each haptic to maintain the IOL position more stably. Significant differences in the IOL tilt, IOL decentration, and postoperative logarithm of the minimum angle of resolution VA were not found between the two groups, which is in accordance with the present study.

The potential complications reported for both techniques are haptic exposure, suture-related complications, elevated intraocular pressure, hypotony, iris capture of the IOL, IOL dislocation, vitreous hemorrhage, and endophthalmitis [3, 17]. One case of haptic migration from the scleral tunnel to the ciliary sulcus occurred in the sutureless intrascleral fixation group; no other complications developed in the current study, but the short follow-up period should be a consideration when analyzing these data. A review by Kumar [18] of 735 cases of sutureless fixation by the glued intraocular lens technique found that haptic-related complications were seen in the late post operative period and included haptic displacement (2.0%), subconjunctival haptic (1.5%) and haptic extrusion (0.5%). According to previous studies, the most common reason for the haptic exposure is the incorrect construction of the scleral tunnel, which may be deep and thick enough to prevent this complication [19]. Although rare, early haptic exposure has been reported, and scleral fragility was also proposed to play a role in this case [20]. It is important to emphasize that IOL fixation techniques should be performed by properly trained vitreoretinal surgeons, minimizing complications.

The study limitations included a comparison of the results of two different surgeons, limited sample size, and short follow-up time. More studies are needed, especially to determine the long-term results of this technique. Further, it is important to better correlate the data regarding the IOL position with the clinical data, best-corrected VA, and postoperative refraction [21]. The anatomic features, i.e., IOL tilt and haptics positions, and not the corrected distance VA and refractive results, were the primary end points because the visual outcomes can be affected by numerous factors, such as preexisting retinal diseases, macular pathologies, corneal astigmatism, preexisting corneal decompensation from multiple surgeries, and even the bias of patient information. However, future reports with larger samples and longer follow- up periods are needed to better analyze the functional results.

## Conclusion

We concluded that, in this study, the sutureless scleral fixation of IOLs was an alternative technique for treating patients with deficient capsular support, and the technique was comparable to the conventional technique with sutures when considering the IOL position and postoperative visual acuity and refraction results.

## Data Availability

The data used and/or analyzed during the current study are available from the corresponding author on reasonable request.

## Abbreviations

UBM: ultrasound biomicroscopy
IOL: intraocular lens
VA: visual acuity
